# Adenosine Deaminase Deficiency – More Than Just an Immunodeficiency

**DOI:** 10.3389/fimmu.2016.00314

**Published:** 2016-08-16

**Authors:** Kathryn V. Whitmore, Hubert B. Gaspar

**Affiliations:** ^1^Molecular and Cellular Immunology Section, UCL Institute of Child Health, University College London, London, UK

**Keywords:** adenosine deaminase, immunodeficiency, SCID, neurological abnormalities, enzyme replacement therapy, hematopoietic stem cell transplantation, gene therapy

## Abstract

Adenosine deaminase (ADA) deficiency is best known as a form of severe combined immunodeficiency (SCID) that results from mutations in the gene encoding ADA. Affected patients present with clinical and immunological manifestations typical of a SCID. Therapies are currently available that can target these immunological disturbances and treated patients show varying degrees of clinical improvement. However, there is now a growing body of evidence that deficiency of ADA has significant impact on non-immunological organ systems. This review will outline the impact of ADA deficiency on various organ systems, starting with the well-understood immunological abnormalities. We will discuss possible pathogenic mechanisms and also highlight ways in which current treatments could be improved. In doing so, we aim to present ADA deficiency as more than an immunodeficiency and suggest that it should be recognized as a systemic metabolic disorder that affects multiple organ systems. Only by fully understanding ADA deficiency and its manifestations in all organ systems can we aim to deliver therapies that will correct all the clinical consequences.

## Introduction

### What Is the Role of Adenosine Deaminase in the Purinergic Signaling Pathway?

**Figure 1:**
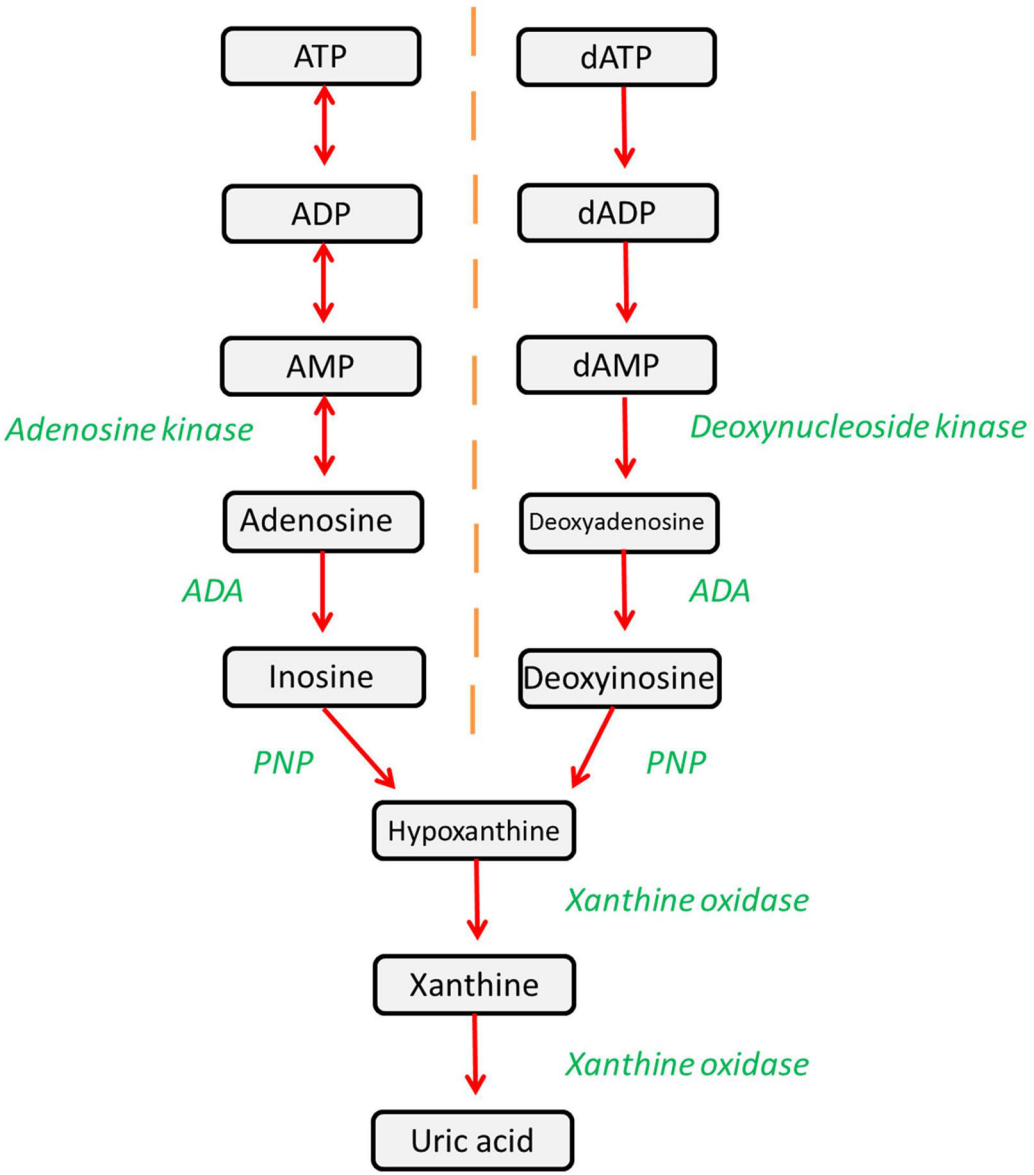
**The purine salvage pathway is an integral metabolic pathway, responsible for the regulation and availability of purines**. ADA catalyzes the deamination of adenosine and deoxyadenosine, forming inosine and deoxyinosine (respectively), which can then undergo further downstream processing. Alternatively, adenosine and deoxyadenosine may be released to activate downstream pathways.

Adenosine deaminase (ADA) is a ubiquitously expressed metabolic enzyme that plays an integral role in numerous cellular processes. It is expressed both intracellularly and, in certain cell types, is also complexed with CD26 on the cell surface. Enzymatically, ADA functions as part of the purine salvage pathway, catalyzing the irreversible deamination of adenosine and deoxyadenosine as shown in Figure 1 (1).

Deficient and impaired ADA activity results in the accumulation of metabolic substrates of which adenosine, deoxyadenosine, and dATP are of particular importance due to their profound effects on cellular function (2). Lymphocyte maturation and function is particularly affected and, hence, deficiency of ADA leads to a severe combined immunodeficiency (SCID) (1). It should be noted that there is an extracellular form of ADA2 that is coded for by the *CECR1* gene (3). Mutations in ADA2 can lead to polyarteritis nodosa and other vasculopathies, and indeed some patients with immunodeficiency have been identified (4–6). However, for the purpose of this review, we will discuss only ADA1 encoded for by the *ADA* gene, since this is the enzyme that is implicated in ADA-SCID, and we will refer to it throughout as ADA.

### What Is ADA-SCID?

Adenosine deaminase deficiency is caused by mutations in the *ADA* gene, and there is a correlation between the severity of the mutation, the level of ADA activity, and the level of metabolic disturbance (7). ADA deficiency is one of the most prevalent forms of SCID and accounts for 15–20% of all cases (1, 8). It is inherited in an autosomal recessive manner and has an overall incidence of ~1:200,000 live births (7). Diagnosis occurs most commonly during infancy and more than 85% of diagnoses occur during the first 6 months of life (7, 9). Initial presentations include recurrent and opportunistic fungal, viral and bacterial infections, lymphopenia, and a failure to thrive (10). Without therapeutic intervention, ADA deficiency has a fatal course due to severe and overwhelming infections and most patients will die within the first year of life (1, 7). Late onset forms are also known, although the manifestations appear to be milder.

### What Are the Treatments for ADA-SCID?

**Figure 2:**
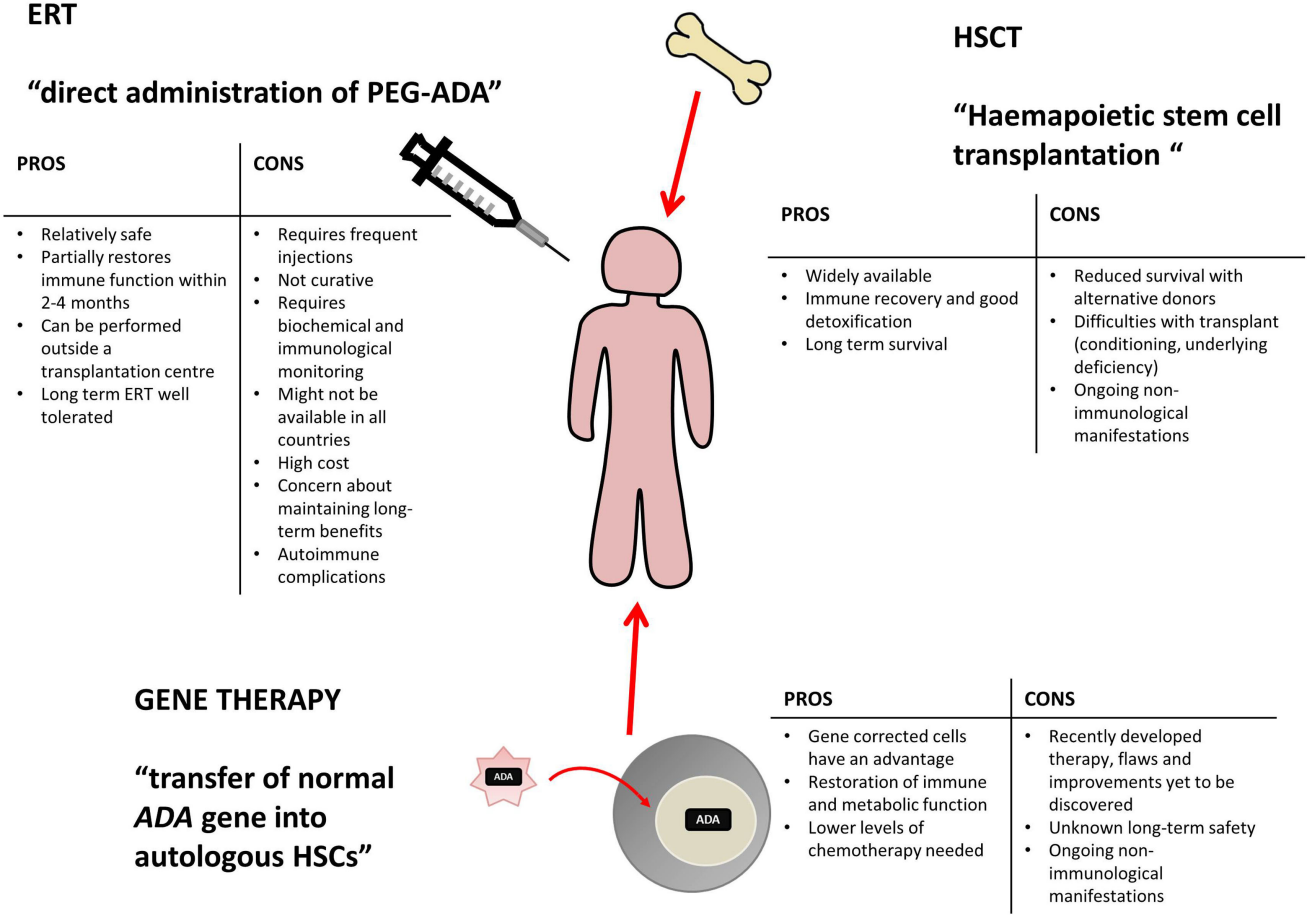
**Potential treatment options for ADA deficiency include enzyme replacement therapy (ERT), hematopoietic stem cell transplantation (HSCT), and gene therapy (GT)**. There are varying benefits and potential problems for each available treatment and patients require individual assessment to decide on the appropriate course of action. This table has been created using information from (1, 10–13).

Various treatment options are currently available for ADA deficiency, as shown in Figure 2, including enzyme replacement therapy (ERT), hematopoietic stem cell transplantation (HSCT, sometimes referred to as bone marrow transplantation), and more recently gene therapy (GT) (10).

Enzyme replacement therapy, HSCT, and GT are all viable treatment options, tailored to individual patient circumstance and treatment availability. Varying levels of immune reconstitution can be achieved by each, and the treatments generally correct the immunological impairment, albeit to different extents (12, 14). HSCT and GT are curative treatment options and tend to be favored, while ERT offers a more immediate treatment option for patients upon initial diagnoses and often acts as a stabilizing measure, while alternative and definitive options are identified (12, 13).

While the focus largely remains on the immunological compartment, a multi-organ pathology can be expected in a metabolic disorder, such as ADA deficiency, because of the ubiquitous nature of ADA expression. Therefore, in this review, we will outline the impact of ADA on various organ systems, starting with the immunological abnormalities. We will also discuss possible pathogenic mechanisms and thereby highlight areas of improvement for current treatments. In doing so, we aim to present ADA deficiency as more than an immunodeficiency – it should be recognized as a systemic metabolic disorder that results in multiple organ pathologies.

## ADA in the Immune System

The metabolic deficiency of ADA principally affects the immune system, and is characterized by severe lymphopenia (affecting T cells, B cells, and NK cells) and impaired cellular and humoral immunity (1, 15). Patients, therefore, present early in life with recurrent infections and a failure to thrive. Current evidence suggests that the immune deficiency is a direct consequence of ADA deficiency, through the accumulation of metabolic substrates and their subsequent downstream effects.

Much research has been conducted to elucidate the mechanism behind the different elements of the immunodeficiency. This section of the review will focus on, in particular, the effect of ADA deficiency on the development of the immune system, the functioning of the T- and B-cell compartments, and the effect on autoimmunity, highlighting important pieces of evidence and discussing proposed mechanisms of pathophysiology.

### Evidence for Abnormal Thymocyte Development and Function

Under normal metabolic circumstances, extremely high levels of ADA enzyme are found within the thymus and, therefore, ADA deficiency will likely lead to an especially devastating effect in this organ (16). In the mouse model, analysis of 3-week-old ADA-deficient mice found that the organs of the immune system (thymus, lymph nodes) were decreased in size and contained fewer cells in comparison to littermate controls (17). The observations made within this Apasov led study are indicative of lymphopenia, which appeared to be progressive in the mice, with more severe lymphopenia being seen at a 3-week analysis compared to earlier time points. Furthermore, although the immune system organs appeared smaller, no changes in levels of apoptosis were detected in the peripheral lymph organs (spleen, lymph nodes), but increased levels of apoptosis were identified in ADA-deficient thymi – the cortical and medullary regions being particularly affected. Evidence of abnormal thymocyte distribution in ADA-deficient mice (specifically a decrease in the number of CD4+ and CD8+ double-positive thymocytes) demonstrates defective thymocyte development (7, 17). This is concurrent with evidence from patients, where severe thymus atrophy and a decrease in thymic dendritic cells are reported (18). However, it is largely unknown which stage of thymocyte development and differentiation is arrested by ADA deficiency. A body of evidence exists to suggest that more differentiated thymocyte populations (double positive, single positive) are significantly reduced in number, most likely due to a developmental or differentiation block at an earlier stage (19). Double-negative thymocytes represent a population early on in the maturation process and are subdivided into four stages (DNI–DNIV). Some evidence suggests that the aforementioned block occurs at the DNIII stage, at a time between the T-cell receptor (TCR)-β and TCR-α gene rearrangements (20). However, contradictory evidence has since suggested that the block in differentiation might not occur at a discrete stage, but is instead due to inhibited proliferation of more mature thymocytes (19). The induction of apoptosis continues to be implicated in the defective thymocyte development (19, 20). Therefore, while there is sufficient evidence to suggest abnormal thymocyte development and differentiation, the exact stage of block remains unknown and research is ongoing.

### Evidence for Abnormal T-Cell Function

Patients with ADA deficiency show a striking T-cell depletion and this is also replicated, albeit to a lesser extent, in the murine model (21). Despite this T-cell lymphopenia, ADA-deficient mice appeared to have a normal distribution of peripheral T cells, although the expression of cell surface markers was altered in comparison to control mice, for example, in both CD4+ and CD8+ ADA-deficient T cells, CD44 and CD69 expression was increased while CD62L expression was decreased (17). The murine model of ADA deficiency additionally shows defective TCR activation *in vivo*, which is suggested to be adenosine mediated (17). T cells from ADA-deficient patients have also proven to be defective in transducing signals through the TCR; however, in patients, it is suggested that it is not adenosine, but 2′deoxyadenosine that exacerbates the defective TCR signaling (18, 22).

### Evidence for Abnormal B-Cell Development and Function

The immunodeficiency associated with ADA deficiency also affects B cells, and patients exhibit severe B-cell lymphopenia and hypogammaglobulinemia. In murine studies led by Aldrich, initial stages of B-cell development occurring in the bone marrow were not shown to be impaired in ADA deficiency (23). Results of this study, therefore, implicate either differentiation within the peripheral lymphoid organs or signaling via the B-cell receptor in the observed B-cell phenotype. The ADA-deficient mice exhibited smaller spleens with reduced cellularity and altered B-cell distribution among marginal, follicular, and newly formed splenic zones (17, 23). Further splenic alterations observed include fewer and smaller germinal centers with abnormal architecture (23). Therefore, from this study, it seems likely that while ADA deficiency does not impair primary B-cell development in the bone marrow, the B-cell defect is due to abnormal differentiation in peripheral lymphoid organs possibly due to an intrinsic B-cell defect or due to the lack of T-cell help. Formation of the germinal center is severely impacted in ADA deficiency, which is likely to affect antigen dependent development of B cells and subsequent generation of B-cell memory. In fact, B lymphocytes in ADA-deficient mice appeared to have reduced proliferative ability, with accumulation of IgM antibodies and concomitant low levels of IgG (23). Furthermore, these B lymphocytes showed defects in activation, with a high tendency to undergo B-cell receptor-mediated apoptosis, potentially caused by the increased levels of dATP detected in the spleen.

Similar results have been shown in untreated ADA-SCID patients, whereby, despite peripheral lymphopenia, only a partial block in B-cell development is demonstrated within the bone marrow (24). On the contrary, untreated ADA-SCID patients displayed a twofold decrease in the number of B cells found in later developmental stages, suggesting that the B-cell defect affects more mature B cells, one hypothesis being that B cells possibly require different levels of ADA during differentiation (24). Furthermore, the importance of ADA to normal B-cell function has been demonstrated by the reduced proliferation of B cells from ADA-SCID patients *in vitro*, despite these patients receiving ERT, and also by the reduction in B-cell activating factor receptor (BAFF-R) in these patients (24, 25). The BAFF-R is expressed on peripheral B cells and it is integral not only in maintaining peripheral B-cell homeostasis but also in determining the number of mature B cells (24, 25).

Therefore, cumulative defects in the typical B-cell response contribute to the B-cell lymphopenia observed in ADA deficiency (23). Existing evidence suggests that ADA deficiency also affects the B-cell compartment in terms of repertoire diversity (26). Eliciting a response to a broad range of antigens is integral to the functioning of the immune system, and V(D)J recombination contributes to the diverse B-cell repertoire (27). It has been demonstrated that accumulating levels of dATP, secondary to ADA deficiency, may affect V(D)J recombination, in terms of the frequency and the nature of specific nucleotide insertions (26).

### Evidence for Autoimmunity

Autoimmunity is a condition commonly associated with some primary immune deficiencies (PID) (28). ADA-SCID represents a PID in which extensive immune dysregulation may disrupt integral adaptive and innate immune pathways that normally lead to the development of tolerance (1). This is especially found in delayed onset ADA phenotypes where there is some evidence of T/B-cell development, which is likely to be abnormal, and also in patients who are reconstituting after PEG-ADA treatment. Furthermore, complete immune reconstitution is not achieved by the available treatments, and this may increase the likelihood of developing autoimmunity (1). The causes of autoimmunity are not well established but may well relate to dysregulated T- and B-cell development and the lack of appropriate regulation.

### Toxic Effect Caused by 2′Deoxyadenosine

2′deoxyadenosine is currently favored as one of the causal factors in the development of immunodeficiency associated with ADA-SCID (16). 2′deoxyadenosine is a component of DNA, and large amounts are generated from DNA degradation (1). Apoptosis is abundant in the thymus, thus providing a large source of DNA for degradation (1, 29). It is likely that the accumulation and uncontrolled elevation of 2′deoxyadenosine, which cannot be metabolized in ADA-deficient patients, exerts lymphotoxic effects either at the nucleoside level or after conversion to dATP by intracellular thymic deoxynucleoside kinase (1, 29).

Acting at the nucleoside level, 2′deoxyadenosine irreversibly inactivates S-adenosyl-l-homocysteine (SAH) hydrolase (16). Inhibition of SAH hydrolase prevents hydrolysis of SAH, and the accumulated SAH inhibits transmethylation reactions, which are necessary for lymphocyte activation (1, 7, 10, 30). This, therefore, severely impacts the immune system.

An additional metabolic pathway affected by ADA deficiency is the increased phosphorylation of 2′deoxyadenosine to form dATP (16). As levels of circulating 2′deoxyadenosine increase, cellular uptake also increases, and 2′deoxyadenosine undergoes intracellular phosphorylation (1). The accumulation of dATP in erythrocytes and lymphocytes is significant – dATP is an inhibitor of ribonucleotide reductase that is necessary for deoxynucleotide synthesis, and its inhibition depletes the intracellular deoxynucleotide pool (1, 7, 16). Hence, inhibition of ribonucleotide reductase impairs DNA synthesis, replication, and repair; processes that are integral to lymphocyte expansion following antigenic challenge (1, 7, 16).

There are also reports that both deoxyadenosine and dATP can have an effect on various apoptotic signaling cascades. It has been reported that deoxyadenosine interacts with p53 apoptotic pathway (22). Studies also suggest that dATP is involved in the loss of cytochrome *c* from the mitochondria and dATP has been implicated in activation of apoptotic protease-activating factor 1 (APAF-1) (7, 31, 32).

### Toxic Effect Caused by Adenosine

**Figure 3:**
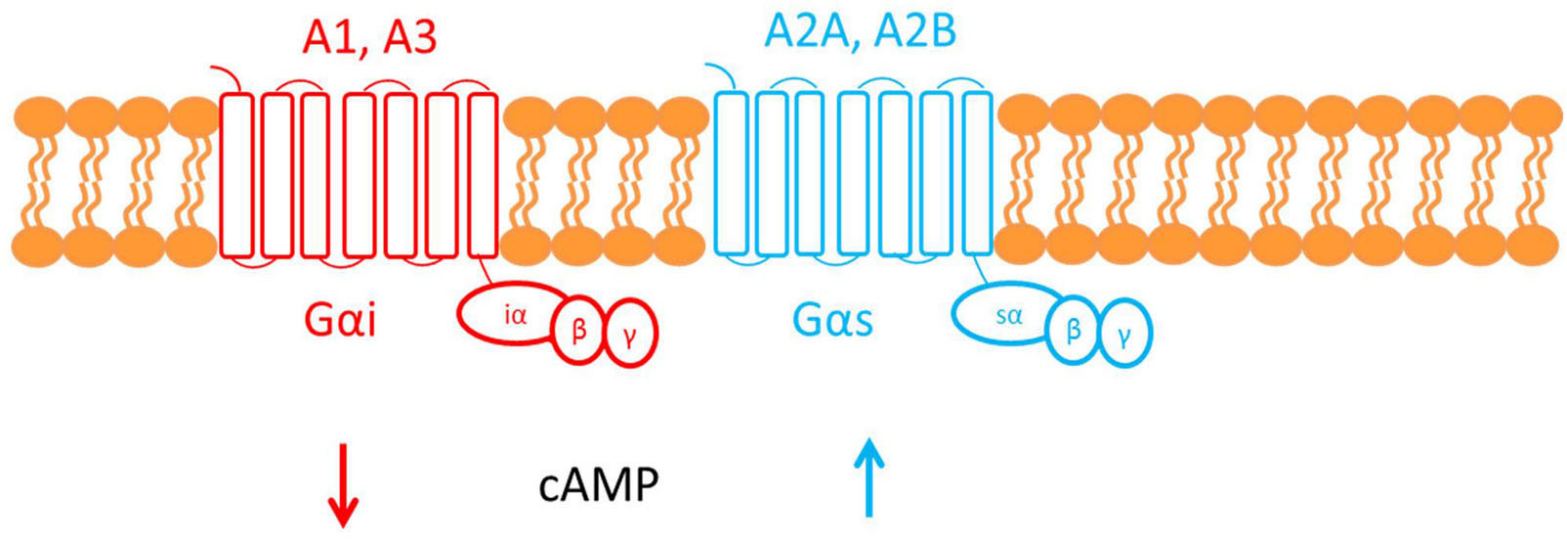
**This figure shows the cell membrane with the adenosine receptors embedded into the phospholipid bilayer**. Adenosine receptors are G-protein-coupled receptors made up of seven transmembrane domains. There are four subtypes of adenosine receptor: A1, A2A, A2B, and A3. The A1 and A3 adenosine receptors are coupled to Gαi, which causes a decrease in cAMP downstream of receptor activation. Conversely, A2A and A2B are both coupled to Gαs, which causes an increase in cAMP.

Adenosine deaminase deficiency leads to accumulation of both 2′deoxyadenosine and adenosine. An alternate hypothesis is that elevated concentrations of adenosine can affect the immune system via activation of its cognate G-protein-coupled receptors (GPCR) found on the plasma membrane of target cells (see Figure 3) (22).

Adenosine, therefore, functions as an extracellular signaling molecule: under normal physiological conditions, it plays a role in fine-tuning the immune response (1). However, this balanced regulation might be disrupted when adenosine concentrations are elevated, as in ADA deficiency. For example, activation of the A2A adenosine receptor by adenosine mediates immunosuppressive actions: cAMP generated downstream of receptor activation acts via a negative feedback loop to inhibit and trigger “off” signaling pathways in activated immune cells (33). It has been suggested that the defective TCR-dependent activation, characteristic in ADA deficiency, may be exacerbated by this adenosine receptor-mediated immunosuppression (1). Investigating studies show that increased cAMP, produced by A2A receptor activation, interferes with signaling events that occur once the TCR have been activated and, therefore, may also inhibit downstream effector functions. The ability of exogenous adenosine to reduce TCR-triggered activation has also been shown both *in vivo* and *in vitro* in CD4+ and CD8+ T cells (22).

**Figure 4:**
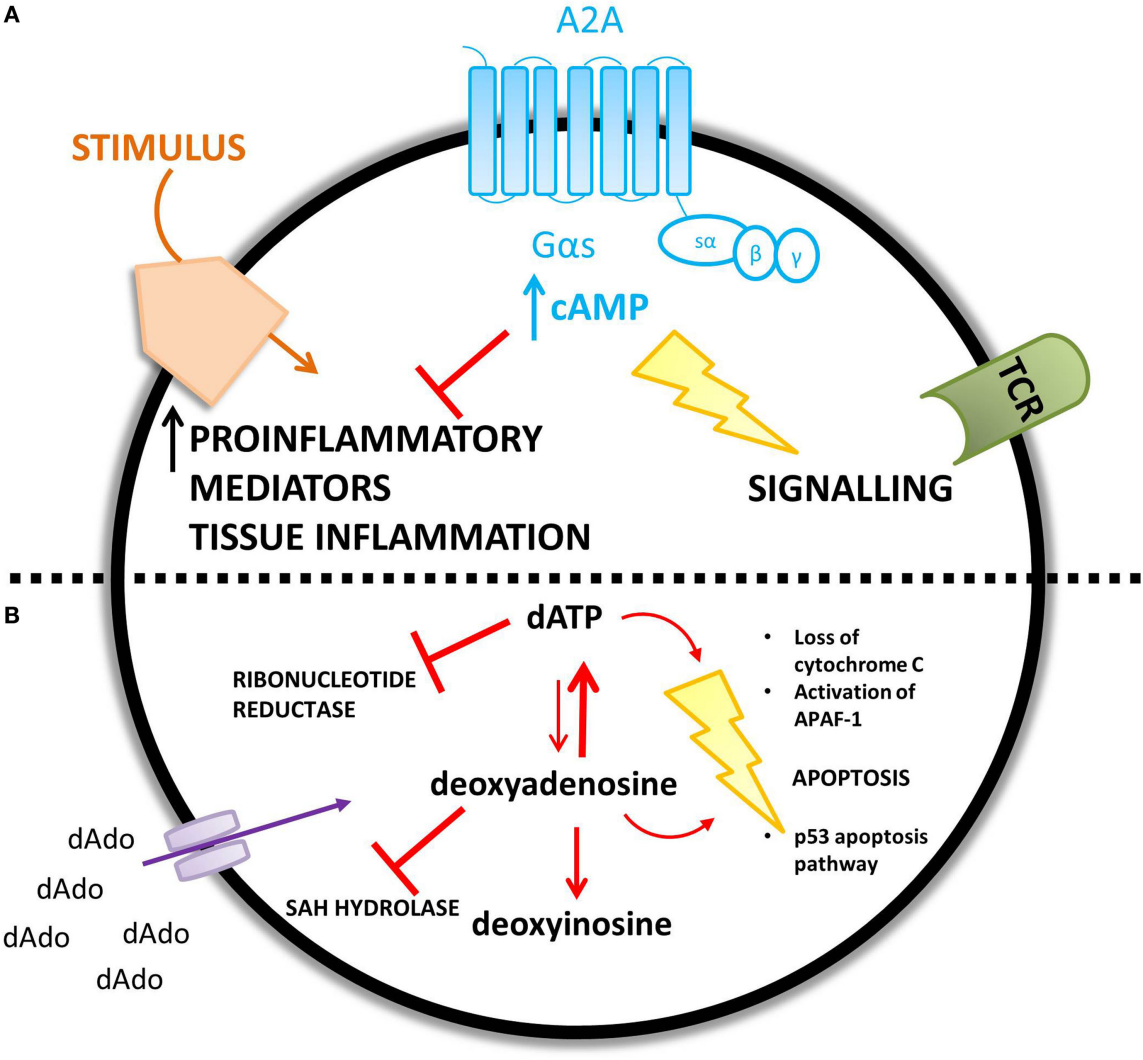
**ADA deficiency leads to an accumulation of adenosine (A) and deoxyadenosine (B) – different mechanisms are proposed for the increased concentration of each metabolic substrate**. **(A)** An increase in extracellular adenosine concentration leads to an increase in intracellular cyclic AMP (cAMP) caused by increased A2A receptor activation. cAMP is proposed to mediate lymphotoxic effects by disrupting TCR signaling and inhibiting the immune response to a stimulus. **(B)** Extracellular accumulation of 2′deoxyadenosine increases the intracellular concentration of 2′deoxyadenosine via diffusion down its concentration gradient. 2′deoxyadenosine inhibits SAH hydrolase and plays a role in apoptosis, by activating the p53 pathway. Alternatively, 2′deoxyadenosine can undergo conversion to dATP. dATP inhibits ribonucleotide reductase and also plays a role in apoptosis.

The proposed mechanisms for the observed immunological abnormalities are summarized in Figure 4.

## ADA in the Brain

**Figure 5:**
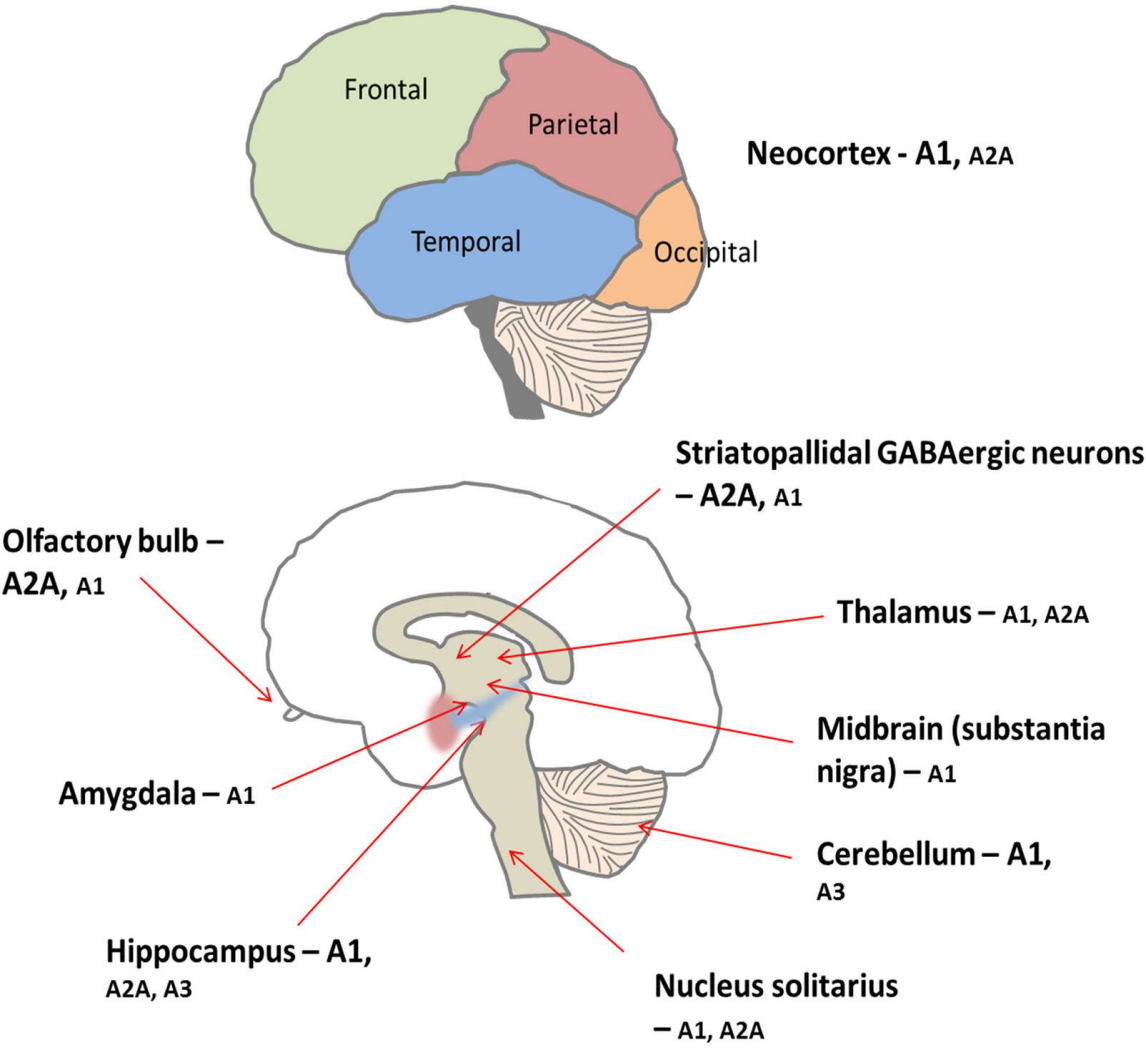
**There is peripheral expression of all four adenosine receptor subtypes in the brain, which are found pre, post, and non-synaptically (34)**. A1 receptors are ubiquitously expressed with high concentrations particularly found in the cortex, hippocampus, and cerebellum (34). Conversely, levels of A2A receptor are generally lower throughout the brain but are concentrated predominantly on medium spiny neurons of the striatum (34). Furthermore, the A1 receptor subtype is preferentially activated by released adenosine, and the A2A receptors are preferentially activated by adenosine formed from adenine nucleotides (35). Low levels of A2B and A3 receptors are found in the brain; these receptors are thought to be involved in pathological situations (35, 36).

Adenosine is ubiquitously expressed, in both the peripheral and central nervous systems (CNS). In the CNS, adenosine functions as a neuromodulator, influencing synaptic transmission and integrating various signaling networks within the brain (34). As in the periphery, there are four subtypes of adenosine receptor in the CNS; the A1 and A2A receptor subtypes predominate, while A3 and A4 are much less prevalent (see Figure 5) (34).

There have been significant advances in the treatment of ADA-SCID, resulting in improved levels of immune reconstitution and, therefore, prolonged survival rates (15). As patients are now surviving for longer, an increasing number of non-immunological manifestations are being identified with cognitive, behavioral, and neurological abnormalities being among the most important of these non-immunological manifestations.

### Evidence for Cognitive and Behavioral Abnormalities

When assessing cognitive ability, SCID patients generally achieve below average IQ scores – however, the level of function is shown to be dependent upon the specific type of SCID (37, 38). Patients with ADA-SCID consistently achieve IQ scores that are not only below the population average but also below that of other SCID groups (37, 38). Furthermore, ADA-SCID patients exhibit hyperactivity, attention deficits, aggressive behavior, and social problems, scoring abnormally in all of these areas of behavior (37). An inverse relationship has been observed between cognitive ability and behavioral difficulties: ADA-deficient patients with lower IQ scores showed increased incidence of emotional and behavioral difficulties (38).

While this evidence seems to implicate ADA deficiency, it has previously been postulated that the observed cognitive and behavioral abnormalities, also present in other forms of SCID, might in fact be a treatment side effect (39). In particular, HSCT is suspected to cause such neurological abnormalities as a side effect (40, 41). However, studies have shown that cognitive and behavioral abnormalities are more frequently observed in ADA-SCID patients than in other SCID groups, despite both cohorts receiving HSCT as treatments (37, 38). Although there are a number of possible causative variables, a body of evidence now suggests that IQ score, and therefore cognitive ability, is perhaps not greatly affected by factors, such as age at transplantation, conditioning regime, and time spent in hospital, and it is, therefore, unlikely that the cognitive abnormalities are caused by the treatment itself (37, 38, 40, 42). Rather, a significant inverse correlation between dATP levels at diagnosis and total IQ has been demonstrated (37). Therefore, it seems likely that deficiency of ADA and the underlying metabolic disturbances are implicated in the establishment and/or development of the observed behavioral and cognitive abnormalities. Interestingly, ADA is a polymorphic enzyme where one of the alloenzymes (ADA1*2) demonstrates a 35% reduction in enzymatic activity (43). The polymorphism involves an Asp to Asn amino acid substitution at position 8 of the mature protein, and data from a particular study suggests that it is more likely to find this rare low-activity polymorphism [ADA-Asn8 (ADA1*2)] in children with mild mental retardation of unknown causes, when compared to both healthy controls and children with moderate to severe mental retardation of known causes (43). Therefore, this extends the theory that deficiency of ADA may be implicated in the cognitive abilities mentioned above, since similar observations are also made in patients with a low-activity ADA polymorphism.

### Evidence for Auditory Abnormalities

A spectrum of auditory abnormalities have been reported in patients with ADA deficiency, in particular, bilateral sensorineural deafness has been investigated as a non-immunological manifestation of ADA deficiency (44, 45). One of the earliest reports of bilateral sensorineural deafness in ADA deficiency describes two siblings who achieved normal immune reconstitution after being successfully treated with HSCT (45). However, severe deafness and mild hearing loss was exhibited in these patients at 1 year of age, with no identifiable genetic-, structural-, drug-, or infection-related cause. Importantly, these children were transplanted without any conditioning and so the deafness could not be attributed to side effects of treatment, thus implicating metabolic perturbation due to ADA deficiency (45).

Incidence of bilateral sensorineural deafness was also investigated in a larger cohort of ADA-SCID patients receiving BMT – a 58% incidence was reported in the 12 patients who had undergone audiologic assessment in a study by Albuquerque and Gaspar (44). This observation was compared against a control group of 16 patients, who had received BMT for immunodeficiencies specifically affecting and confined to the immune system, and it was found that in this control cohort only one child displayed bilateral sensorineural hearing loss (6% incidence). No audiologic assessments were undertaken prior to transplantation, and it is, therefore, not known whether these auditory abnormalities existed before treatment. However, deafness is not a complication typically associated with BMT, and this evidence, therefore, supports the hypothesis that auditory abnormalities are not a complication from treatment, but are instead likely to be another non-immunologic manifestation of ADA deficiency. Although there was no visible relationship between severity of deafness and dATP levels, affected patients also showed no identifiable predisposing factors; therefore, the role of ADA deficiency in auditory abnormalities needs to be investigated and further defined (44).

### Evidence for Structural Abnormalities Linked to Motor Abnormalities

While there is clear evidence that patients with ADA deficiency are susceptible to neurocognitive abnormalities, very little research has been done to explore the effect on the brain structurally and sub-structurally. One investigation reported by Nofech-Mozes studied three ADA-deficient patients who presented with neurological abnormalities (such as seizures, developmental delay, hypotonia) with no other identifiable etiology (for example, infection or transplant-related medication toxicity). Cranial MRI and computed tomographic (CT) scans showed that these patients exhibited volume loss and abnormalities, particularly affecting the basal ganglia and thalamus (46). Therefore, it is possible that the deficiency of ADA, or the accumulation of metabolic substrates, is a possible cause for the neurological abnormalities observed in this patient cohort (see Table 1).

**Table 1:** **A case report investigating neurological abnormalities in patients with ADA deficiency emphasizes the implication of such abnormalities**.

Age of first neurological abnormality	Initial neurological presentation	Neurological abnormalities (pre- and post- treatment)	Treatment	Outcome
4 months	Smiling ceased and vocalization was reduced, was unable to make eye contact	Observed pre-treatment: dilation of ventricular system and pericerebral fluid spaces	Initial supportive care followed by BMT at 9 months	Little neurologic improvement before BMT
	Displayed hypotonia of trunk and extremities			Death from respiratory insufficiency and multi-organ failure at 11.5 months
	Displayed head lag and severe rotary nystagmus			
3 months	Moderate hypotonia with head lag	Observed pre-treatment: caudate nuclei, basal ganglia, and ventral thalami show cystic changes, calcification, and volume loss	BMT at 4 months	Immune reconstitution after treatment
				Developed generalized seizures at 5 months
				No leukocytes/lymphocytes in CSF
				Severe psychomotor retardation at 12 months, with hypotonia of all extremities
				Non-verbal, poor swallowing coordination and no progression in motor or cognitive skills at 6 years
4 months	Moderate hypotonia with head lag	*Observed pre-treatment*: prominent pericerebral fluid and ventriculomegaly – suggestive of extraventribular obstructive hydrocephalus	HSCT at 5 months	12-, 18-, 24-, 36-, and 48-month assessments showed severe sensory-neural deafness and moderate–severe motor and cognitive psychomotor delay
		*Observed post-treatment*: slight resolution of pericerebral fluid, but persistence of ventriculomegaly		Developed generalized seizures at 2 years

*Patients present with neurological manifestations early on in life. This evidence is significant because it identifies structural changes in the brains of these patients, possibly caused by ADA deficiency. It also highlights that existing treatments failed to correct and/or prevent progression of the abnormalities. This table was created using data from Nofech-Mozes et al. (46)*.

*BMT, bone marrow transplantation; HSCT, hematopoietic stem cell transplantation; CSF, cerebrospinal fluid*.

Therefore, the evidence presented so far suggests that ADA deficiency is somehow associated with the observed neurological abnormalities in this metabolic disorder. In fact, neurological abnormalities are far more prevalent in ADA-SCID than other forms of immunodeficiency, indicating that the underlying metabolic disruption caused by ADA deficiency affects the CNS, both in function and development. It is not known what degree of metabolic detoxification is achieved in the CNS with current ADA treatments, nor is it known whether neurological abnormalities are present prior to treatment. Evidence provided by multiple papers seems to suggest that current treatments are not sufficient to prevent the development of and/or correct these neurological abnormalities long term (2, 37, 38, 44–46). A study investigating CNS outcomes in ADA-deficient patients after HSCT found that 6 out of 12 patients, at a mean follow-up period of 12 years, showed distinct neurological abnormalities, despite displaying complete immune reconstitution (2). Elements of all the neurological abnormalities reported above (cognitive and behavioral abnormalities, auditory abnormalities, and motor abnormalities) are reported in multiple patients within this long-term study. Furthermore, this paper presents patients who have received transplants both with and without conditioning, therefore reiterating previous statements that the neurological abnormalities are not treatment-related (2). In addition to the display of neurocognitive abnormalities, ADA-deficient patients display abnormalities in the brain architecture, such as the ventriculomegaly described above (46).

Current research has not, so far, investigated the mechanism of pathology. One possibility is that the accumulation of dATP causes neurotoxic effects by a similar mechanism elucidated in the immune system. To investigate this concept, levels of deoxyadenosine and dATP would need to be studied before and after various treatments in all ADA-deficient patients, regardless of the presence of neurological abnormalities. However, pre- and post-measurement of specific parameters, such as cognitive ability, may be difficult due to the young age of patients at the time of diagnosis and/or at treatment (38).

Alternatively, another possible hypothesis is that the high concentrations of adenosine could be activating adenosine receptors. Adenosine is identified as a neuromodulator; therefore, it is fair to assume that altering levels of neuromodulation will have a significant effect within the brain. Adenosine receptors are found in particular abundance in the basal ganglia and thalamus, and changes in finely tuned synaptic transmission within these brain regions may explain some of the neurological abnormalities reported in ADA deficiency. Furthermore, there are an array of other disease states in which adenosine is pathologically implicated, including but not limited to Alzheimer’s disease, schizophrenia, epilepsy, and pain (35). Therefore, if adenosine is implicated in the pathogenesis of other diseases, it is possible that alterations in adenosine metabolism occurring in ADA deficiency are affecting the functioning of the CNS, resulting in the neurological abnormalities reported in many patients.

Therefore, this highlights the importance of elucidating the mechanisms underlying these neurological abnormalities in ADA deficiency. This knowledge will aid in the development of improved therapies that are capable of correcting and/or preventing neurological abnormalities from occurring in patients. It is also important to fully understand the function of ADA and the purine salvage pathway in the brain, not only to correct the abnormalities seen in ADA deficiency but also to improve therapies for other neurological disorders in which adenosine is pathologically implicated. This is particularly relevant in the case of PNP deficiency that is another PID resulting from defects in the purine salvage pathway and, interestingly, a high proportion of patients present with neurological abnormalities similar to those described in ADA deficiency (47, 48).

## ADA in the Hepatic System

Establishing a model system for disease states is integral to fully understanding the underlying molecular mechanisms. An ADA-deficient murine model currently exists that replicates many of the phenotypes exhibited in patients. However, the successes of early attempts to establish a representative mouse model were hindered due to adverse effects of ADA deficiency on the hepatic system, which were not typically associated with patients. This section will look at the effect of ADA deficiency on the hepatic system of animals and patients.

### Evidence in Animals

An ADA-deficient murine model was developed by two independent groups who, despite targeting different sites of the gene, both successfully developed ADA-deficient mice displaying similar phenotypes (21). However, the majority of homozygous mice were either stillborn or succumbed within the first 2 days postpartum (49). While the exact time point was unclear, it was estimated that a narrow window of death existed around the time of parturition (49, 50). Histological analysis showed normal lymphoid tissues in the majority of ADA-deficient mice, but changes in liver, small intestine, and lung were revealed (49, 50). The liver was particularly affected – hepatocytes appeared morphologically altered, and the affected number increased with gestational age, hence indicating that the metabolic disorder underlying ADA deficiency caused pathology in the murine liver (50). Further investigation revealed marked changes in the levels of adenosine and deoxyadenosine in the liver, which were barely detectable in control and heterozygous mice but were found at extremely elevated levels in ADA-deficient mice (49). Additionally, levels of dAXP were higher in deficient mice and there was a reduction in activity levels of SAH hydrolase (49). In summary, ADA-deficient mice exhibited severe hepatocyte impairment and degeneration that proved perinatally lethal.

### Evidence in Patients

It is evident that ADA plays an integral role in the liver during fetal development of the murine model. However, the effects in ADA-deficient patients are not perinatally lethal and hepatic dysfunction has rarely been reported. For example, in one particular report, a neonatal ADA-SCID patient exhibited hyperbilirubinemia and hepatitis; but both pathologies were successfully resolved following treatment with ERT (51). Additional clinical findings include histological abnormalities and liver stiffness alongside fatty liver and/or hepatomegaly, with abnormalities appearing to resolve alongside ERT (52). However, other supportive clinical data are hard to find.

In conclusion, there is evidence for hepatic abnormalities in the murine model and, to a much lesser extent, ADA-deficient patients. ADA-deficient mice exhibited severe hepatocyte impairment and degeneration that proved to be perinatally lethal. The severe pathology exhibited suggests that the mother was incapable of detoxifying the fetal metabolic load caused by ADA deficiency: indeed, accumulation of metabolic substrates has been demonstrated experimentally (49, 50). One hypothesis is that adenosine kinase (ADK), suggested to be the main pathway of deoxyadenosine phosphorylation in mice, is abundant in the liver and, therefore, dATP accumulation is likely and may cause the hepatic abnormalities (50). The accumulation of adenosine shows a 30- to 100-fold increase and could also be the cause of hepatic damage via adenosine receptor activation (49). Akin to the immune system, deoxyadenosine mediates inhibition of SAH hydrolase and, therefore, the accumulation caused by ADA deficiency may be responsible for reduced activity.

Therefore, it is possible that accumulation of a combination of metabolic substrates causes perinatally lethal hepatic damage in ADA-deficient mice. While this lethality is not mimicked in patients, ADA is still ubiquitously expressed and hepatic abnormalities should not be overlooked as a possible non-immunological manifestation. In fact, it has been stated that elevated serum transaminase levels may be seen in patients with ADA deficiency: this is indicative of liver damage and often etiology is unknown (49). Therefore, although patient data are limited, there is sufficient murine evidence to suggest that ADA deficiency can largely impact upon the liver and, therefore, hepatic abnormalities may possibly occur in patients due to metabolic disruption and, thus, warrants further investigation.

## ADA in the Pulmonary System

As previously mentioned, homozygous ADA knockout mice of the initial murine model died either before or shortly after birth, seemingly due to consequences of metabolic disorder. In order to overcome the perinatal lethality caused by ADA deficiency, genetically engineered mice were developed in which ADA activity was restored to the trophoblast cells only (21, 53). Therefore, mice no longer succumbed perinatally and postnatally exhibited multiple phenotypes of ADA deficiency, including pulmonary insufficiency (53).

### Evidence in Animals

These newly modified ADA-deficient mice survived past birth, but instead died from severe respiratory distress: initial symptoms of tachypnea were observed around day 12 postpartum and progressed until days 19–25, when mice succumbed (53). Upon further investigation, ADA-deficient mice exhibited severe pulmonary inflammation; the airways also appeared to have undergone remodeling and were hyperresponsive (see Table 2) (21, 53).

**Table 2:** **Pulmonary abnormalities observed in ADA-deficient mice include pulmonary inflammation and airway remodeling, shown by various pathological changes (53, 54)**.

Tissue damage and airway remodeling	Pulmonary inflammation
↑ Size of alveolar spaces ↑ Thickness of pulmonary blood vessel smooth muscle	↑ Number of inflammatory cells: Eosinophil infiltration Large and foamy macrophages
Hypertrophy of bronchial epithelium	Alveolar macrophages engulfing eosinophils in BALF
↑ Mucus production	Accumulation of activated alveolar macrophages and eosinophils
Occlusion of airways with cellular debris	

*BALF, bronchoalveolar lavage fluid*.

In order to ascertain whether the observed phenotype was related to the deficiency of ADA, the effect of ERT on the lung abnormalities was investigated (53, 54). PEG-ADA was administered to the ADA-deficient mice at day 18, by which point the pulmonary phenotype was fully established (53). An initial decrease in eosinophils was monitored, and a further decrease in alveolar macrophages occurred after 2 weeks of ERT. This study also observed a reduction in mucus production in the enzyme-treated mice, although an improvement in alveolar enlargement was not observed. However, since this observation on alveolar enlargement was made after 72 h, it could be possible that an improvement might be seen after a longer time period. This study, therefore, demonstrates that particular elements of both pulmonary inflammation and airway remodeling appear to be reversed by ERT (53). It was also reported that treated mice demonstrated significantly lower levels of respiratory distress and remained alive while maintained on therapy. This evidence indicates that the observed pulmonary abnormalities are likely to be caused by the deficiency of ADA. Moreover, a decrease in both adenosine and 2′-deoxyadenosine levels were measured in PEG-ADA treated mice (53).

A more recent paper describes development of pulmonary fibrosis in ADA-deficient mice (55). ADA-deficient mice were treated with varying concentrations of PEG-ADA, and the effect of changing endogenous adenosine concentrations in the lungs was monitored. High adenosine concentrations in the lung (achieved by low dosage ERT) resulted in pulmonary inflammation and airway remodeling (as seen in previous reports), but also resulted in the expression of profibrotic molecules and collagen deposition – these are characteristic indicators of pulmonary fibrosis. In fact, it was demonstrated that reducing adenosine levels after the initial onset of pulmonary fibrosis was sufficient to resolve the established fibrosis (55).

Another pulmonary phenotype observed in ADA-deficient mice is pulmonary alveolar proteinosis (PAP), a rare lung disorder that can result in progressive respiratory failure. This has been identified in mice, in addition to the pulmonary inflammation and airway restructuring seen previously (56).

Therefore, it is evident that ADA and its role in metabolism has significant consequences for the murine respiratory system in a multitude of ways.

### Evidence in Patients

Patients with ADA deficiency present with severe lung abnormalities; however, this is not unique to ADA-SCID, and lung abnormalities are present in other variants of SCID (57). Nevertheless, there is substantial evidence that the development of lung abnormalities in ADA deficiency is not infection related and, rather, likely to be derived from metabolic insufficiency (57). This study also demonstrated that while patients with two different forms of SCID (ADA and X-linked) both exhibit similar radiological and respiratory findings, positive microbiology from respiratory cultures is significantly less frequent in ADA-deficient patients. Therefore, it is highly likely that patients with ADA deficiency develop non-infective lung abnormalities. Histological analysis of these non-infective lung lesions in symptomatic patients has shown that the abnormalities are highly suggestive of PAP (57, 58). Therefore, ADA-deficient patients exhibit similar pulmonary phenotypes to those observed in the murine model (58). The incidence of ADA-deficient patients with PAP was reported to be 43.8% in this Grunebaum study, where 7/16 patients showed characteristic lung pathology indicative of PAP that was not observed in patients with other forms of SCID (0/22). Further comparisons of ADA patients showed that there was no significant difference in clinical and immunological characteristics between those diagnosed with PAP and not diagnosed with PAP and, therefore, the pathology is likely to be a non-immunological abnormality resulting from ADA deficiency. Furthermore, it was found that treatment for ADA deficiency (including PEG-ADA or HSCT) appeared to correct the pulmonary complications: all patients (but one) showed rapid resolution of PAP (58).

A recently emerging technique (impulse oscillometry) was employed to measure lung function via a method alternative to spirometry, and this also highlighted a high incidence of airway dysfunction in ADA-SCID patients (59). Furthermore, use of this methodology within this study demonstrated that peripheral airway dysfunction is still present in a significant fraction of ADA-SCID patients post-treatment (specifically ERT and GT). Not only does this add weight to the hypothesis that perturbations in adenosine metabolism are linked to the development of pulmonary abnormalities in ADA deficiency, but it also suggests that current therapies may not be sufficiently controlling and/or preventing pulmonary abnormalities in ADA deficiency.

## ADA in the Skeletal System

From the cumulative evidence so far, it is clear that ADA deficiency has a multi-organ system pathology. Bone defects are radiologically detectable in ~50% of patients with early-onset ADA deficiency (60). However, bone defects are also observed in other immunodeficiencies and, therefore, it is unclear whether the bone abnormalities exhibited in ADA deficiency are a result of the perturbations in purinergic metabolism or secondary to the immunodeficiency (60).

### Evidence in Animals

Adenosine deaminase-deficient mice exhibit bony abnormalities, including rib cage curvature and enlarged costochondral junctions (21). Similar bony phenotypes are seen between ADA-deficient mice and Rag2γc^−/−^ mice (another immunodeficiency) (60). However, this Sauer led study showed that in fact some bone abnormalities, such as low bone mass, were observed only in ADA-deficient mice, therefore implying that at least some of the displayed bony phenotype must be a direct consequence of ADA deficiency. Investigating the ADA-deficient bone phenotype more extensively showed reduced bone formation resulting from decreased osteoclastogenesis and impaired osteoblast (OBs) function. Additionally, this study also showed the bone marrow microenvironment to be defective in its ability to support hematopoiesis in ADA-deficient mice. Furthermore, neonatal treatment with ERT, HSCT, and GT in these mice can lead to correction of the altered bone phenotype (60).

### Evidence in Patients

It is common for patients with ADA deficiency to display costochondral abnormalities and skeletal dysplasia (60). An autopsy report of ADA-SCID patients found short growth plates, within which only a few proliferating and some hypertrophic chondrocytes were found (61). This autopsy also revealed necrotic chondrocytes and high levels of cellular debris in these patients. However, other immunodeficiencies also display bone abnormalities, and bony pathology that is supposedly characteristic to ADA-SCID has been found in patients with normal ADA activity, albeit to a lesser extent (60, 62). Therefore, it is unknown whether these bony defects are caused by the disrupted purine metabolism or the underlying immunodeficiency (10, 60). However, since there seems to be a bony phenotype found more specifically among ADA deficiency, it can be hypothesized that the bony abnormalities may also be formed independently of the immunodeficiency (60).

Osteoblasts and osteoclasts (OCs) are two important cell types found within the bone, responsible for formation and resorption of bone, respectively, and therefore interaction between the two is critical for maintenance of bone homeostasis (60). RANKL and its decoy receptor OPG are responsible for mediating cross-talk between OBs and OCs, and the RANKL:OPG ratio regulates the formation and activity of OCs. Therefore, RANKL and OPG play important roles within the bone environment. OBs are a source of RANKL, but RANKL can also be produced by activated T and B cells. Similarly, B cells within the bone marrow are a major source of bone marrow OPG. Therefore, since ADA deficiency is characterized by T- and B-cell lymphopenia, the consequent effect of this on the RANKL/OPG axis implicates an immune-dependent process that can lead to bony abnormalities. OBs and OCs both express adenosine receptors, and therefore abnormal adenosine signaling as a result of increased adenosine concentrations may also play an important role in modulating their activity and be a causal factor in the development of bone abnormalities in ADA deficiency (60).

An additional hypothesis suggested is that the OB defect may play another contributory role in the bony abnormalities observed in both ADA-deficient patients and the murine model (60). Wild-type OBs have high ADA enzymatic activity, but there is a threefold reduction in activity in mesenchymal progenitor cells, from which OBs originate and suggests that OB differentiation must involve upregulation of ADA expression. Hence, since OBs become increasingly dependent on ADA during proliferation and differentiation, ADA deficiency could potentially have a severe impact on the viability and function of OBs. This hypothesis is explored *in vitro*, whereby lentiviral vector transduction with functional ADA gene is sufficient to correct the growth defects seen in ADA-deficient OBs (60).

Therefore, bony abnormalities do exist in ADA deficiency, and these appear, in part, to be a non-immunological manifestation and not entirely secondary to the immunodeficiency. This ought to be taken into consideration in the treatment of ADA deficiency; all three treatments of ERT, BMT, and GT achieve near-complete corrections of bone abnormalities in mice, yet this efficiency is not entirely replicated in patients. It has been suggested that ERT may be less efficient in patients, since mice receive a high dosage of PEG-ADA at an earlier time point than patients typically receive treatment (60).

Therefore, a full understanding of the immune-dependent and immune-independent pathogenesis might lead to improved therapy and correction of bony abnormalities.

## ADA in the Renal System

While reports of renal abnormalities in ADA deficiency are not in abundance, there is still sufficient evidence to link ADA deficiency with kidney dysfunction. Adenosine is known to play an integral and diverse role in kidney function and, therefore, this system may be susceptible to alterations in purine metabolism caused by ADA deficiency (63).

### Evidence in Mice

Renal abnormalities have been noted in ADA-deficient mice, although in comparison to other organ systems, this appears not to have been fully investigated. Early reports on the ADA-deficient mouse model noted abnormalities in the kidneys, including a change in appearance. There was also a large increase in number of red blood cells present in both the glomeruli and convoluted tubules (21). Similar observations were noted in a paper investigating chronic kidney disease (64). This group employed the ADA mouse model to demonstrate renal dysfunction and fibrosis: mice receiving a low dose regimen of ERT, thus modeling ADA deficiency, exhibited significant vascular damage and fibrosis in the glomeruli and interstitial tissues. These renal abnormalities appeared to be prevented when deficient mice were maintained on a high dosage PEG-ADA regimen.

### Evidence in Patients

Initial observations from an autopsy report of ADA patients revealed renal abnormalities including: mesangial sclerosis in seven cases, with two showing global sclerosis in 5–10% of glomeruli (61). Increased mesangial matrix was additionally seen in three cases. A recent publication provides further evidence linking ADA deficiency with renal dysfunction: hemolytic uremic syndrome (HUS) is a common cause of renal failure in young children, and this manifestation is reported in a cohort of ADA-SCID patients (9). Coincidentally, ERT led to successful stabilization of renal function in two of these patients in this study.

Adenosine deaminase is ubiquitously expressed, and despite the scarcity of evidence about renal abnormalities caused by ADA deficiency, it would be an oversight to assume the underlying metabolic disturbance caused by ADA deficiency does not affect renal function. Therefore, further research is required to investigate this concept.

Similar to other non-immunologic manifestations, the underlying mechanism of renal pathology is not known. However, research is being focused on the role of adenosine signaling on kidney function. Dai et al. investigated a link between A2B adenosine receptor activation (caused by increased renal adenosine) and renal dysfunction, not only in the ADA-deficient mice but also in two other mouse models. Specifically, this group identifies the induction of IL-6 downstream of A2B adenosine receptor activation as a contributing candidate for the observed renal fibrosis, although it has also been demonstrated that mice deficient in IL-6 are not protected from developing renal fibrosis (64, 65).

However, it is possible that adenosine signaling may play a dual role within the renal system: it seems that under conditions where adenosine concentrations are persistently elevated, as in ADA deficiency, A2B receptor signaling can promote renal fibrosis. On the other hand, it does also appear that short-term activation of the A2A and A2B adenosine receptors can decrease inflammation and reduce fibrosis (65). ADA-deficient mice are shown to display increased levels of A2B receptor expression, and perhaps this avenue warrants further investigation to allow us to distinguish between the protective and harmful role of adenosine signaling (64).

Similar to the case in mice, adenosine-mediated receptor activation has again been implicated in patients – downstream actions in the renal system include constriction of afferent renal arterioles and reduction in glomerular filtration via A1 adenosine receptor activation (9). Although this would not explain the diagnosis of HUS, it may provide an explanation for the severe renal failure exhibited by ADA-deficient patients, and the subsequently poor clinical response (9).

## ADA and Skin Tumors

Dermatofibrosarcoma protuberans (DFSP) is a mesenchymal tumor of the skin and its incidence in the general population is 4.2 per million. One study describes the occurrence of this rare skin tumor in 12 unselected patients with ADA deficiency (66). The frequency of this observation suggests that ADA patients have a susceptibility to this particular tumor. The mechanism is unclear, but, given that some tumors developed after successful HSCT or ERT, a lack of tumor surveillance is unlikely, although a very specific defect cannot be excluded. It was also suggested that high levels of tissue adenosine could predispose to a more fibrogenic dermal environment that, when coupled to an increased rate of single-strand DNA breaks caused by elevated deoxyadenosine, could lead a more tumorigenic predisposition in the skin of ADA-deficient individuals. Again, this requires further monitoring and investigation.

## Conclusion

Adenosine deaminase deficiency is a SCID that manifests itself in multiple organ systems. Treatment is essential and life-saving, yet improvements still need to be made to ensure the systemic multi-organ pathology is corrected. Since ADA deficiency is a metabolic disorder, substrate accumulation is assumed to underlie the pathogenesis. However, to a large extent, it is still relatively unknown by which mechanism this occurs. Having elucidated the mechanisms within the immune system, deoxyadenosine may act in a similarly toxic manner in other organ systems and may be additionally responsible for the non-immunological manifestations. Alternatively, the impact of adenosine and its downstream signaling pathway following receptor activation should also be considered.

Therefore, having clearly laid out the evidence for ADA deficiency as a multi-organ system disease, it is important to further investigate the mechanisms underlying the different organ manifestations in order to improve existing treatments and allow a more complete correction.

## Author Contributions

KW and HG equally helped develop the concept, prepare the manuscript, and draft and finalize the final version.

## Conflict of Interest Statement

HG is founder and Chief Scientific Officer of Orchard Therapeutics and holds equity in the company. KW declare that the research was conducted in the absence of any commercial or financial relationships that could be construed as a potential conflict of interest.

## Disclaimer

The endorsement of this manuscript by the reviewer RS reflect the views of the reviewer and should not be construed to represent the views or policies of the FDA.
